# The Relationship between Teacher Support and Students' Academic Emotions: A Meta-Analysis

**DOI:** 10.3389/fpsyg.2017.02288

**Published:** 2018-01-22

**Authors:** Hao Lei, Yunhuo Cui, Ming Ming Chiu

**Affiliations:** ^1^Institute of Curriculum and Instruction, East China Normal University, Shanghai, China; ^2^Department of Special Education and Counselling, The Education University of Hong Kong, Hong Kong, Hong Kong

**Keywords:** teacher support, academic emotions, meta-analysis, students, moderator analysis

## Abstract

This meta-analysis examines the association between teacher support and students' academic emotions [both positive academic emotions (PAEs) and negative academic emotions (NAEs)] and explores how student characteristics moderate these relationships. We identified 65 primary studies with 58,368 students. The results provided strong evidence linking teacher support and students' academic emotions. Furthermore, students' culture, age, and gender moderated these links. The correlation between teacher support and PAEs was stronger for Western European and American students than for East Asian students, while the correlation between teacher support and NAEs was stronger for East Asian students than for Western European and American students. Also, the correlation between teacher support and PAEs was strong among university students and weaker among middle school students, compared to other students. The correlation between teacher support and NAEs was stronger for middle school students and for female students, compared to other students.

## Introduction

As students spend much of their time with their teachers in school, teacher support can be vital to students' academic development, including not only learning outcomes but also affective or emotional outcomes. Many empirical studies have shown that teacher support was significantly positively correlated with positive academic emotions (PAEs; e.g., enjoyment, interest, hope, pride, and relief) and significantly negatively correlated with negative academic emotions (NAEs; anxiety, depression, shame, anger, worry, boredom, and hopelessness), but their effect sizes vary substantially across studies (Skinner et al., 2008; Mitchell and DellaMattera, 2011; King et al., 2012; McMahon et al., 2013; Liu et al., 2016). Hence, there is a need for a systematic integration of the results of these studies to better understand the relation between teacher support and students' academic emotions and attributes that moderate this relation. This meta-analysis addresses this issue by examining 65 primary studies with 58,368 students. We begin by defining the two central notions: teacher support and academic emotions.

### Teacher support

Self-determination and social support offer two definitions for *teacher support*. The self-determination view suggests that teacher support occurs when students perceive cognitive (Skinner et al., 2008), emotional (Skinner and Belmont, 1993), or autonomy-oriented support from a teacher during the students' learning process (Wellborn and Connell, 1987). According to Ryan and Deci (2000), individuals do work and complete tasks based on their values, interests, and hobbies, but others close to them can influence their related emotions and motivations. Teacher support includes three dimensions: support for autonomy, structure, and involvement. Support for autonomy is teacher provision of choice, relevance, or respect to students. Structure is clarity of expectations and contingencies. Involvement is warmth, affection, dedication of resources, understanding the student, or dependability (Skinner et al., 2008). Research applying this definition of teacher support has found that it can influence anxiety, depression, hope, and other emotions among students (Reddy et al., 2003; Skinner et al., 2008; Van Ryzin et al., 2009).

In the social support model, teacher support can be viewed in two ways: broad or narrow. The broad perspective, based on Tardy's (1985) social support framework, defines teacher support as a teacher giving informational, instrumental, emotional, or appraisal support to a student, in any environment (Tardy, 1985; Kerres Malecki and Kilpatrick Demary, 2002). Informational support is giving advice or information in a particular content area. Instrumental support is giving resources such as money or time. Emotional support is love, trust, or empathy. Appraisal support is giving evaluative feedback to each student (Malecki and Elliott, 1999). The narrow perspective views teacher support in the form of help, trust, friendship, and interest only in a classroom environment (Fraser, 1998; Aldridge et al., 1999).

Teacher support enhances a teacher's relationship with a student. Specifically, teachers who support students show their care and concern for their students, so these students often reciprocate this concern and respect for the teacher by adhering to classroom norms (Chiu and Chow, 2011; Longobardi et al., 2016). When teachers shout at students, blame them, or aggressively discipline them, these students often show less concern for their teachers and fewer cooperative classroom behaviors (Miller et al., 2000).

As might be expected from this variation and diffuseness in definitions of teacher support, none of them specify a direct relationship between teacher support and students' academic emotions, making it difficult to determine the salient levers for intervention and support. Therefore, we conduct a meta-analysis to integrate these diverse frameworks and streamline the knowledge base, thereby promoting the development of this field.

### Academic emotions

*Academic emotions* refer to the emotional experience of learning (and teaching), including enjoyment, hopelessness, boredom, anxiety, and anger (Pekrun et al., 2002), which can affect students' learning outcomes (Dong and Yu, 2007). Researchers have generally divided academic emotions into two categories: positive academic emotions (PAEs) and negative academic emotions (NAEs); however, they disagree about how to delineate their boundaries. According to Pekrun et al. (2002), PAEs include relief, hope, enjoyment, and pride, while NAEs include shame, anxiety, boredom, anger, and hopelessness. Other researchers also include calmness and contentment in PAEs or depression and fatigue in NAEs (Dong and Yu, 2007; Sorić, 2007). PAEs may also include excitement, happiness, and other indicators (Dong and Yu, 2007), while NAEs may include sense of threat, fear, and others (Dong and Yu, 2007). Based on the literature, the current study define PAEs as including interest, hope, enjoyment, pride, calmness, contentment, and relief; and NAEs as including shame, anxiety, anger, worry, boredom, depression, fatigue, and hopelessness. For a fuller picture, the measurement of academic emotions should include both PAEs and NAEs.

### The relationship between teacher support and students' academic emotions

Many empirical studies have shown that students with more teacher support have higher PAEs or lower NAEs. Specifically, students with more teacher support have more enjoyment, interest, hope, pride, or relief (PAEs); or less anxiety, depression, shame, anger, worry, boredom, or hopelessness (NAEs) (Ahmed et al., 2010; King et al., 2012; Tian et al., 2013). As the effect sizes differ substantially among these studies (Skinner et al., 2008; King et al., 2012; McMahon et al., 2013; Liu et al., 2016), later studies tried to summarize the earlier results (e.g., Weber et al., 2001; Clark, 2008; Arbeau et al., 2010; Lazarides and Ittel, 2013). However, these studies only partly verified the underlying phenomena, as some studies had limitations such as convenience sampling or ignoring sample size –resulting in low reliability and reducing the quality of the research. Therefore, to determine clearly the link between teacher support and students' academic emotions, a meta-analysis is needed.

Through a review of past empirical research on teacher support and students' academic emotions, we found that many effect sizes were heterogeneous, suggesting that moderators might account for these differences. Specifically, we examined the potential moderating roles of students' cultures, ages, and genders.

### Potential moderators of the link between teacher support and students' academic emotions

#### Culture

Several studies have implied that culture may influence the association between teacher support and students' academic emotions. For example, Karagiannidis et al. (2015) study of students from Greece showed a strong correlation between teacher support and PAE indicators but only a weak correlation between teacher support and NAE indicators. In contrast, King et al.'s (2012) study of students from Philippines, found a weak correlation between teacher support and PAE indicators but a strong one between teacher support and NAE indicators.

#### Age

The link between teacher support and students' academic emotions might differ by the latter's (Klem and Connell, 2004; Frenzel et al., 2007). For example, past studies found that the relation between teacher support and indicators of PAE was lowest among middle school students and highest among university students, relative to elementary and high school students (Aldridge et al., 2013; Liu et al., 2016). Meanwhile the link between teacher support and indicators of NAE was strongest for middle school students (Taylor, 2003; Huang et al., 2010; Martínez et al., 2011). According to these findings, we expect age to moderate the relation between teacher support and students' academic emotions.

#### Gender

Female students tend to receive more teacher support than do male students (Lutz, 1996; Baumeister and Sommer, 1997), and several empirical studies have shown gender differences in the link between teacher support and indicators of students' academic emotions, such as interest, depression, anxiety (Van Ryzin et al., 2009; Sylva et al., 2012; Nilsen et al., 2013). According to these findings, we expect gender to moderate the correlation between teacher support and students' academic emotions.

### Study purpose

This meta-analysis of 65 studies analyzed the relations between teacher support and students' academic emotions (positive and negative) and their moderators. Specifically, this study examined: (a) the correlations between teacher support and students' positive academic emotions, (b) the correlations between teacher support and students' positive academic emotions, and (c) whether culture, age, or gender moderated these correlations.

## Methods

### Literature search

To locate studies on teacher support and students' academic emotions, we systematically searched the literature from January 1994 (Through search in above-mentioned database, “the relationship between teacher support and students' academic emotions” was firstly proposed by Karabenick and Sharma, 1994) to January 2016 using the following electronic databases: ProQuest Dissertations, Web of Science, Google Scholar, Springer, Taylor & Francis, EBSCO, PsycINFO, and Elsevier SDOL. Indexed keywords constituted terms reflecting teacher support (*support, involvement, care*/*caring, warmth, closeness, teacher enthusiasm, teacher help, learning environment, classroom environment, social support, relationship between teacher and student/child*) and academic emotions (*anxiety, pride, shame, achievement emotion, interest, anger, depression, enjoyment, boredom, hope, worry, hopelessness, positive affect, academic emotions, negative affect, relief*, *well-being*). We obtained full-text versions of articles from libraries when they could not be found online, limiting ourselves to articles written in English. We used inclusion and exclusion criteria described in the next subsections to analyze and filter the collected studies.

### Literature exclusion criteria

We included articles based on the following criteria: (a) studying the relationship between teacher support and students' academic emotions, (b) measuring teacher support, including any of the keywords mentioned above, (c) measuring academic emotions, again including any of those above keywords, (d) including an explicit sample size, and (e) explicitly reporting the Pearson product-moment correlation coefficient (*r*) or a *t* or *F* value that could be transformed into *r*. After applying the inclusion and exclusion criteria, 65 articles remained.

### Coding

To facilitate meta-analysis, feature coding was conducted on 65 articles. We considered the following variables: author(s) and publication year, proportion of male students, ages, indicators of teacher support, indicators of academic emotions, types of academic emotions (PAEs and NAEs), number of students, and *r* effect size. The following criteria guided the coding procedure (see Table 1): (a) effect sizes of each independent sample were coded based on an independent sample, and separately coded if a study had several independent samples; (b) correlations between different indicators of teacher support and academic emotions were separately coded; (c) correlations between teacher support and different indicators of academic emotions were separately coded; (d) this number was used if an independent sample provided effect sizes (expressed as *r*) for sample characteristics such as sex; and (e) if a study reported multiple correlations between teacher support and an academic emotion, their mean value was used.

**Table 1:** Studies included in the meta-analysis.

**Author (year)**	***N***	***r***	**TS indicator^a^**	**AE indicator^b^**	**AE type**	**Culture^c^**	**Age^d^**	**Male (%)^e^**
Afari, 2013	352	0.24	TS	Enjoyment	PAEs	1	4	0.34
Ahmed et al., 2010	238	0.28	TS	Interest	PAEs	2	2	0.46
Ahmed et al., 2010	238	0.45	TS	Enjoyment	PAEs	2	2	0.46
Ahmed et al., 2010	238	−0.21	TS	Anxiety	NAEs	2	2	0.46
Aldridge et al., 2013	352	0.24	TS	Enjoyment	PAEs	3	4	0.656
Allen and Fraser, 2007	520	0.21	TS(p)	Enjoyment	PAEs	2	1	0.5
Allen and Fraser, 2007	520	0.01	TS(s)	Enjoyment	PAEs	2	1	0.5
Arbeau et al., 2010	169	−0.26	C	Anxiety	NAEs	2	1	0.499
Arslan, 2009	466	−0.21	TS	Anger	NAEs	3	3	0.457
Birgani et al., 2015	180	0.36	TS	Enjoyment	PAEs	3	3	1
Burić, 2015	365	0.27	TS	Enjoyment	PAEs	3	3	0.356
Burić, 2015	365	0.22	TS	Hope	PAEs	3	3	0.356
Burić, 2015	365	0.19	TS	Pride	PAEs	3	3	0.356
Burić, 2015	365	0.15	TS	Relief	PAEs	3	3	0.356
Burić, 2015	365	−0.17	TS	Anger	NAEs	3	3	0.356
Burić, 2015	365	−0.06	TS	Anxiety	NAEs	3	3	0.356
Burić, 2015	365	−0.09	TS	Shame	NAEs	3	3	0.356
Burić, 2015	365	−0.06	TS	Hopelessness	NAEs	3	3	0.356
Cheung, 1995	128	−0.28	TS	Depression	NAEs	1	5	0.475
Chirkov and Ryan, 2001	116	−0.14	TS	Depression	NAEs	2	3	0.422
Chirkov and Ryan, 2001	120	0.08	TS	Depression	NAEs	2	2	0.358
Chirkov and Ryan, 2001	119	0.22	TS	Positively emotions	PAEs	2	2	0.532
Chirkov and Ryan, 2001	118	−0.03	TS	Negatively emotions	NAEs	2	2	0.532
Cox et al., 2009	411	0.45	TS	Enjoyment	PAEs	2	2	0.436
Cox et al., 2009	411	−0.16	TS	Worry	NAEs	2	2	0.436
Demaray et al., 2005	82	−0.04	TS	Emotional symptoms	NAEs	2	2	0.354
Elmelid et al., 2015	643	−0.05	TS	Depression	NAEs	2	5	0.455
Elmelid et al., 2015	643	0.17	TS	Anxiety	NAEs	2	5	0.455
Elmelid et al., 2015	643	−0.06	TS	Depression	NAEs	2	5	0.455
Elmelid et al., 2015	643	0.2	TS	Anxiety	NAEs	2	5	0.455
Federici and Skaalvik, 2014	309	−0.14	ES	Anxiety	NAEs	2	2	0.482
Federici and Skaalvik, 2014	309	−0.13	IS	Anxiety	NAEs	2	2	0.482
Frenzel et al., 2009	1,542	0.48	TE	Enjoyment	PAEs	2	2	0.48
Gläser-Zikuda and Fuß, 2008	431	−0.26	TC	Anxiety	NAEs	2	2	0.494
Hagenauer and Hascher, 2010	356	0.51	TC	Enjoyment	PAEs	2	2	0.336
Hill et al., 1996	87	−0.27	TS	Anxiety	NAEs	2	1	0.471
Huang et al., 2010	158	−0.26	TS	Anxiety	NAEs	1	4	0.684
Jia et al., 2009	706	−0.27	TS	Depression	NAEs	1	2	0.495
Jia et al., 2009	709	−0.25	TS	Depression	NAEs	2	2	0.482
Karabenick and Sharma, 1994	288	−0.11	TS	Anxiety	NAEs	2	4	0.36
Karabenick and Sharma, 1994	288	−0.17	TS	Negatively affect	NAEs	2	4	0.36
Karagiannidis et al., 2015	627	0.47	TS	Enjoyment	PAEs	2	2	0.499
Karagiannidis et al., 2015	627	−0.29	TS	Boredom	NAEs	2	2	0.499
King et al., 2012	1,147	0.15	TS	Enjoyment	PAEs	1	2	0.542
King et al., 2012	1,147	0.12	TS	Hope	PAEs	1	2	0.542
King et al., 2012	1,147	0.07	TS	Pride	PAEs	1	2	0.542
King et al., 2012	1,147	−0.4	TS	Anger	NAEs	1	2	0.542
King et al., 2012	1,147	−0.18	TS	Anxiety	NAEs	1	2	0.542
King et al., 2012	1,147	−0.23	TS	Shame	NAEs	1	2	0.542
King et al., 2012	1,147	−0.47	TS	Boredom	NAEs	1	2	0.542
King et al., 2012	1,147	−0.33	TS	Hopelessness	PAEs	1	2	0.542
Lapointe et al., 2005	593	−0.11	TH	Anxiety	NAEs	2	2	0.496
LaRusso et al., 2008	476	−0.2	TS	Depression	NAEs	2	3	N
Lazarides and Ittel, 2013	212	0.47	TS	Interest	PAEs	2	2	1
Lazarides and Ittel, 2013	149	0.42	TS	Interest	PAEs	2	2	0
Liu et al., 2016	873	0.45	TS	Affect in school	PAEs	1	1	N
Liu et al., 2016	675	0.35	TS	Affect in school	PAEs	1	2	N
Liu et al., 2016	610	0.33	TS	Affect in school	PAEs	1	3	N
Ludwig and Warren, 2009	175	0.43	TS	Hope	PAEs	2	3	0.486
MacPhail, 2012	125	0.25	TS	Positively affect	PAEs	2	1	0.472
MacPhail, 2012	125	−0.18	TS	Negatively affect	NAEs	2	1	0.472
Martínez et al., 2011	140	−0.27	TS	Depression	NAEs	3	2	0.429
Martínez et al., 2011	140	−0.44	TS	Depression	NAEs	3	1	0.429
McMahon et al., 2013	188	0.02	TS	Fear	NAEs	2	5	0.37
McMahon et al., 2013	188	0.09	TS	Worry	NAEs	2	5	0.37
McMahon et al., 2013	188	−0.02	TS	Anxiety	NAEs	2	5	0.37
McMahon et al., 2013	188	−0.18	TS	Depression	NAEs	2	5	0.37
Murberg and Bru, 2009	198	−0.19	TS	Depression	NAEs	2	3	0.439
Myint and Fisher, 2001	1,188	0.31	TS	Enjoyment	PAEs	1	2	0.457
Neville, 2008	159	0.27	TS	Positively affect	PAEs	2	2	0.47
Neville, 2008	159	−0.13	TS	Negatively affect	NAEs	2	2	0.47
Nilsen et al., 2013	319	−0.09	TS	Depression	NAEs	2	5	0.404
Nilsen et al., 2013	319	−0.2	TS	Depression	NAEs	2	5	0.404
Ommundsen and Kvalø, 2007	194	0.63	TS	Enjoyment/Interest	PAEs	2	3	0.515
Ommundsen et al., 2006	760	0.11	TS	Enjoyment	PAEs	2	5	0.499
Pan, 2014	462	0.59	TS	Enjoyment	PAEs	1	3	0.561
Piechurska-Kuciel, 2011	354	−0.78	TS	Anxiety	NAEs	2	2	0.362
Reddy et al., 2003	1,285	−0.28	TS	Depression	NAEs	2	2	1
Reddy et al., 2003	1,300	−0.25	TS	Depression	NAEs	2	2	0
Rey et al., 2007	89	−0.07	TS	Anxiety	NAEs	2	1	0.472
Rueger et al., 2008	108	−0.25	TS	Anxiety	NAEs	2	2	1
Rueger et al., 2008	108	−0.29	TS	Depression	NAEs	2	2	1
Rueger et al., 2008	138	−0.06	TS	Anxious	NAEs	2	2	0
Rueger et al., 2008	138	−0.23	TS	Depression	NAEs	2	2	0
Ryan et al., 2005	474	0.4	TS	Positively affect	PAEs	2	1	0.5
Ryan et al., 2005	474	−0.15	TS	Anxiety	NAEs	2	1	0.5
Sahaghi et al., 2015	180	0.37	TS	Enjoyment	PAEs	3	3	1
Sakiz, 2012	227	0.64	TS	Enjoyment	PAEs	3	4	0.374
Sakiz, 2012	227	−0.55	TS	Hopelessness	NAEs	3	4	0.374
Sakiz, 2012	138	0.6	TS	Enjoyment	PAEs	3	1	0.514
Sakiz, 2012	138	−0.21	TS	Anxiety	NAEs	3	1	0.514
Sakiz et al., 2012	317	0.62	TS	Enjoyment	PAEs	2	2	0.4
Sakiz et al., 2012	317	−0.41	TS	Hopelessness	NAEs	2	2	0.4
Sakiz et al., 2007	99	0.67	TS	Enjoyment	PAEs	2	2	0.343
Sakiz et al., 2007	99	−0.36	TS	Hopelessness	NAEs	2	2	0.343
Skinner et al., 2008	805	−0.1	TS(t)	Bored	NAEs	2	5	0.488
Skinner et al., 2008	805	−0.12	TS(t)	Anxiety	NAEs	2	5	0.488
Skinner et al., 2008	805	−0.1	TS(t)	Frustrated	NAEs	2	5	0.488
Skinner et al., 2008	805	−0.56	TS(s)	Bored	NAEs	2	5	0.488
Skinner et al., 2008	805	−0.36	TS(s)	Anxiety	NAEs	2	5	0.488
Skinner et al., 2008	805	−0.38	TS(s)	Frustrated	NAEs	2	5	0.488
Sun and Hui, 2007	680	−0.28	TS	Depression	NAEs	1	2	1
Sun and Hui, 2007	678	−0.27	TS	Depression	NAEs	1	2	0
Sun et al., 2006	433	−0.26	TS	Depression	NAEs	1	5	0.552
Sylva et al., 2012	1,766	0.53	TS	Enjoyment	PAEs	2	5	0.481
Sylva et al., 2012	1,766	−0.13	TS	Anxiety	NAEs	2	5	0.481
Tanigawa et al., 2011	239	−0.37	TS	Depression	NAEs	2	2	1
Tanigawa et al., 2011	305	−0.36	TS	Depression	NAEs	2	2	0
Taylor and Fraser, 2003	745	−0.04	TS	Anxiety	NAEs	2	3	N
Telan, 2000	694	−0.07	TS	Depression	NAEs	2	1	0.487
Tian et al., 2013	361	0.42	TS	Positively emotions	PAEs	1	5	0.468
Tian et al., 2013	361	−0.31	TS	Negatively emotions	NAEs	1	5	0.468
Van Ryzin, 2011	423	0.25	TS	Hope	PAEs	2	2	0.533
Van Ryzin et al., 2009	231	0.22	TS	Hope	PAEs	2	2	0.524
Wang, 2009	1,042	−0.09	TS	Depression	NAEs	2	2	0.48
Wang and Eccles, 2013	1,157	0.24	TS	Positively emotions	PAEs	2	2	0.48
Way et al., 2007	1,451	−0.26	TS	Depression	NAEs	2	2	0.458
Weber et al., 2001	46	0.14	TS	Affect	PAEs	2	2	0.522
Weber et al., 2001	46	0.12	TS	Affect	PAEs	2	2	0.522
Wentzel, 1998	167	0.39	TS	Interest	PAEs	2	2	0.509
Yang et al., 2015	472	0.53	TS	Positively motions	PAEs	1	1	0.663

a *TS, teacher support; TC, teacher's care; TE, teacher enthusiasm; ET, emotions support; IS, instrumental support; C, closeness; TH, teacher help; (p), parents report; (t), teacher report; (s), students self-report, Others were students self-report*.

b *AE, Academic emotions*.

c *1, East Asia; 2, Western European/American; 3, other*.

d *1, Elementary; 2, Middle School; 3, High School; 4, University; 5, Mixed*.

e *N, Not report*.

When coding was complete, effect sizes between teacher support and students' academic emotions were calculated for each sample, based on the principles of meta-analysis (Lipsey and Wilson, 2001). The moderators tested for influence on the association between teacher support and students' academic emotions were (a) culture, (b) age, and (c) gender.

*Culture* was coded as “East Asia,” “Western European/American,” or “other”; “East Asia” referred to students from Asian countries such as China (including Hong Kong and Taiwan), South Korea, the Philippines, Singapore, and so on. “Western European/American” referred to students from European and North American countries such as Germany, the United States of America, and so on. “Other” referred to students from Turkey, the United Arab Emirates, Iran, and so on. Age was coded as “elementary,” “middle school,” “high school,” “university,” and “mixed.” “Mixed” denoted that the participants in a study included at least two categories of the above school categories. Gender was coded as the proportion of male students.

### Data analysis

We used the comprehensive meta-analysis software CMA 2.0 to analyze all the data. A fixed-effects model calculated the homogeneity and mean effects. Averaged weighted correlation coefficients (within- and between- inverse-variance weights) of independent samples were used to compute mean effect sizes. Moderators were identified by the homogeneity test, which revealed variance in effect sizes between different samples' characteristics. Where the homogeneity test was significant (*Q*_*Bet*_ > 0.05), post-hoc analysis confirmed the different groups statistically. For continuous variables, this study used meta-analysis to examine variation in effect sizes explained by the moderator.

## Results

### Correlation between teacher support and academic emotions

After filtering the literature, we used 65 independent samples, and the sizes of 121 effects were calculated (45 effect sizes between teacher support and PAEs, 76 between teacher support and NAEs). In all, 58,368 students participated in the studies reviewed; sample sizes of individual studies ranged from 46 to 1,766.

To test our hypotheses, we calculated sample sizes (*k*), weighted effect sizes (*r*), and 95% confidence intervals (see Table 2). A fixed effects model was used to homogenize the analysis. The results showed that students with more teacher support had higher PAEs [*r* = 0.340 (*z* = 51.909, *p* < 0.001, *k* = 45, 95% CI = 0.328, 0.351)] or lower NAEs [*r* = −0.215 (*z* = −41.769, *p* < 0.001, *k* = 76, 95% CI = −0.225, −0.206)]. These effect sizes were suitable for moderator analysis (Cohen, 1969).

**Table 2:** Fixed model of correlations between teacher support and academic emotions.

	***k***	***N***	**Mean *r***	**95% CI for** ***r***		**Homogeneity test**			**Tau-squared**			**Test of null (two-tailed)**
				**LL**	**UL**	***Q*(*r*)**	***p***	**I-squared**	**Tau-squared**	**SE**	**Tau**	***z*-value**
PAEs	45	21690	0.340	0.328	0.351	823.197	0.00	94.655	0.038	0.011	0.194	51.909^***^
NAEs	76	36678	−0.215	−0.225	−0.206	1218.358	0.00	93.844	0.032	0.007	0.179	−41.769^***^

**** *p < 0.001*.

### Moderator analysis

To test the aforementioned factors moderating the relationship between teacher support and students' academic emotions, we conducted two total homogeneity tests across 45 and 76 independent samples for PAEs and NAEs respectively. The results showed significant homogeneity coefficients between teacher support and academic emotions (*Q*_*T*__(44)PAE_ = 823.197, *p* < 0.001; *Q*_*T*__(75)NAE_ = 1218.358, *p* < 0.001). This indicates that culture, age, and gender moderated the relations between teacher support and students' PAEs and NAEs. We used meta-analysis of variance to confirm whether culture and age moderated the correlations between teacher support and academic emotions, and used meta-regression analyses to examine whether gender influenced these correlations.

#### Culture

As indicated in Table 3, the homogeneity test showed a significant homogeneity coefficient between teacher support and PAEs across our three cultures (East Asian, Western European/American, and other) (Q_BET_ = 60.599, *df* = 2, *p* < 0.001). As the table shows, the Western European/American group had a stronger correlation (*r* = 0.384, 95% CI = 0.368, 0.400) than the East Asian group (*r* = 0.286, 95% CI = 0.266, 0.305). Likewise, the homogeneity test found significant differences in the correlation between teacher support and NAEs across the three cultures (QBET = 119.523, *df* = 2, *p* < 0.001); however, in this case, the East Asian group (*r* = −0.307, 95% CI = −0.326, −0.288) showed a stronger correlation between teacher support and NAEs than the West European/American group (*r* = −0.190, 95% CI = −0.202, −0.178).

**Table 3:** Culture and age as moderators of the association between teacher support and academic emotions.

	**Between-group effect (*Q_*BET*_*)**	***k***	***N***	**Mean *r***	**SE**	**95% CI for** ***r***		**Homogeneity test within each group (Q_W_)**
						**LL**	**UL**	
**PAEs**								
***Culture***	60.599^***^							
East Asian		11	8434	0.286	0.017	0.266	0.305	270.216^***^
Western European/American		26	11071	0.384	0.016	0.368	0.400	409.543^***^
Other		8	2185	0.315	0.025	0.276	0.352	82.839^***^
***Age***	42.450^***^							
Elementary		7	3122	0.348	0.034	0.316	0.378	133.060^***^
Middle school		23	11841	0.310	0.014	0.294	0.327	378.373^***^
High school		10	3261	0.350	0.020	0.319	0.379	111.512^***^
University		2	579	0.415	0.186	0.346	0.481	10.586^***^
Mixed		3	2887	0.419	0.085	0.388	0.449	121.869^***^
**NAEs**								
***Culture***	119.523^***^							
East Asian		12	8879	−0.307	0.005	−0.326	−0.288	89.629^***^
Western European/American		54	24876	−0.190	0.009	−0.202	−0.178	879.848^***^
Other		10	2923	−0.145	025	−0.181	−0.109	129.358^***^
***Age***	164.830^***^							
Elementary		8	1916	−0.160	0.010	−0.204	−0.116	23.240^*^
Middle school		34	18929	−0.276	0.007	−0.289	−0.262	435.489^***^
High school		9	3461	−0.120	0.003	−0.152	−0.086	16.424^*^
University		5	1313	−0.135	0.075	−0.187	−0.081	105.465^***^
Mixed		20	11059	−0.158	0.018	−176	−0.140	95.982^****^

* *p < 0.05*,

*** *p < 0.001*.

#### Age

The results of the homogeneity test (Q_BET_ = 42.450, *df* = 4, *p* < 0.001) suggested that age influenced the link between teacher support and PAEs. Teacher support was significantly correlated with PAEs for elementary school (*r* = 0.348, 95% CI = 0.316, 0.378), middle school (*r* = 0.310, 95% CI = 0.294, 0.327), high school (*r* = 0.350, 95% CI = 0.319, 0.379), and university (*r* = 0.415, 95% CI = 0.346, 0.481); however, undergraduates showed a stronger correlation than the other students, and middle school students showed a weaker correlation than the other students. As shown in Table 3, the homogeneity test (QBET = 164.830, *df* = 4, *p* < 0.001) suggested that age moderated the link between teacher support and NAEs. Broken down by age group, significant correlations were observed between teacher support and NAEs for elementary students (*r* = −0.160, 95% CI = −0.204, −0.116), middle school students (*r* = −0.276, 95% CI = −0.289, −0.262), high school students (*r* = −0.120, 95% CI = −0.120, −0.086), and undergraduates (*r* = −0.135, 95% CI = −0.187, −0.081). The results indicated that middle school students had a stronger correlation between teacher support and NAEs than the other three groups.

#### Gender

To examine whether gender moderated the link between teacher support and students' academic emotions, r was meta-regressed onto the percentage of male students in each sample. As seen in Table 4, the meta-regression analysis (*Q*_*Model*_[1, *k* = 40]_PAE_ = 0.781, *p* > 0.05) suggested that gender did not moderate the relationship between teacher support and PAEs. However, meta-regression (*Q*_*Model*_[1, *k* = 72]_NAE_ = 4.208, *p* < 0.05) demonstrated that the relation between teacher support and NAEs was moderated by gender; the effect size of the correlation between teacher support and NAEs for an all-female sample (*r* = −0.252) was much stronger than for an all-male sample (*r* = −0.196).

**Table 4:** Meta-regression analyses of gender.

	**Variable**	**Parameter**	**Estimate**	**SE**	***z*-value**	**95% CI for** ***b***	
						**LL**	**UL**
PAEs	Male (%)	β_0_	0.375	0.031	11.961	0.314	0.436
		β_1_	−0.055	0.062	−0.884	−0.176	−0.066
		*Q _*Model*_*(1, *k* = 40) = 0.781, *p* > 0.05					
NAEs	Male (%)	β_0_	−0.196	0.014	−13.859	−0.224	−0.168
		β_1_	0.056	0.027	−2.051	−0.110	−0.002
		*Q _*Model*_*(1, *k* = 72) = 4.208, *p* < 0.05					

### Publication bias

**Figure 1:**
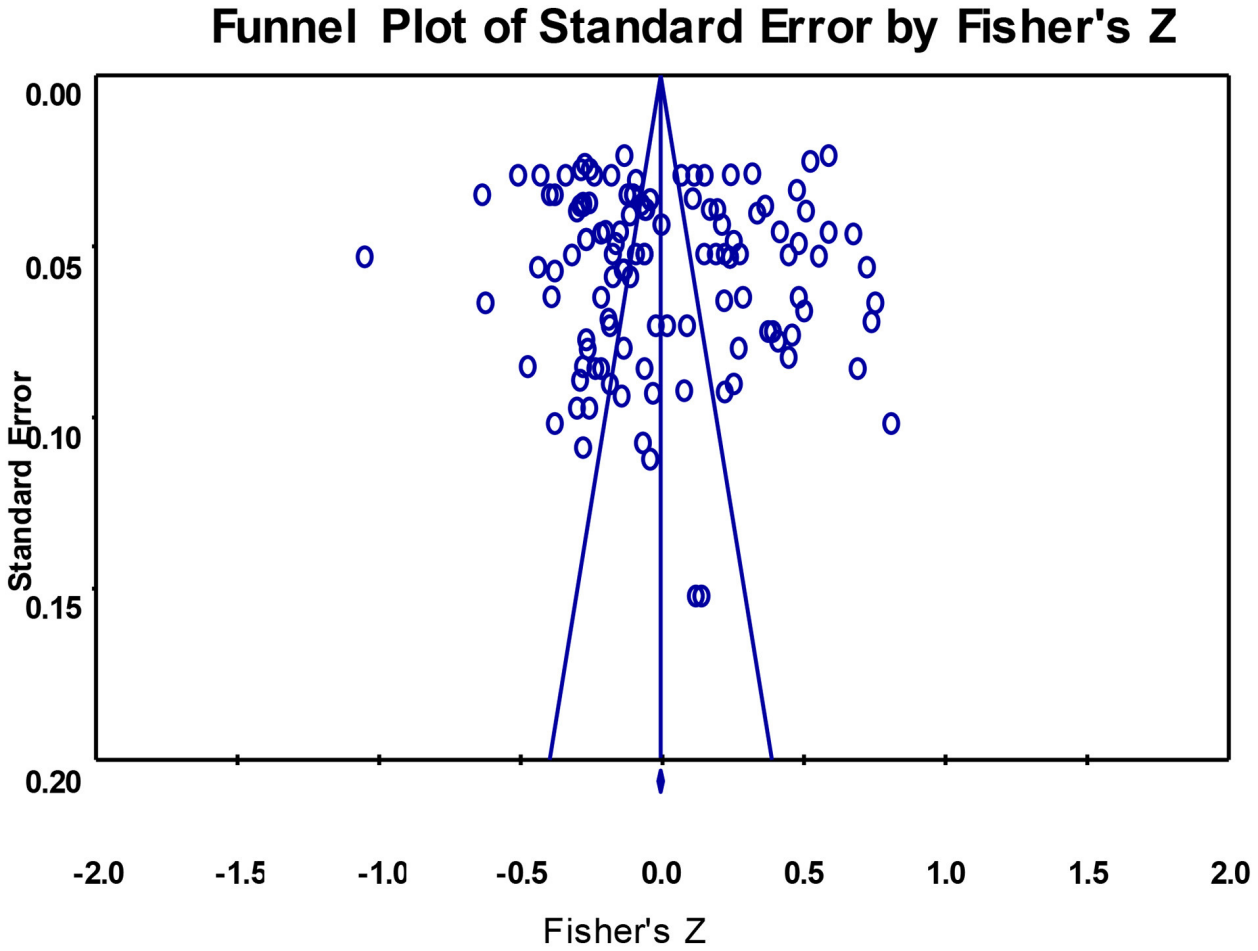
Funnel plot of effect sizes of the correlation between teacher support and academic emotions.

To examine whether the results were biased due to the effect sizes from various sources, a funnel plot was drawn (see Figure 1); it indicated that the 121 effects were symmetrically distributed on both sides of the average in terms of size. Egger's regression (Egger et al., 1997), an effective method for examining publication bias (Teng et al., 2015), revealed no significant bias [*t*_(119)_ = 0.698, *p* = 0.486]. In addition, we also twice conducted Egger's regression analysis on teacher support, for PAEs and for NAEs. The results showed no publication bias [*t*_*PAE*__(43)_ = 0.800, *p* = 0.428; *t*_*NAE*__(74)_ = 0.453, *p* = 0.652]. This indicates that the overall correlation between teacher support and students' academic emotions was stable.

## Discussion

In the current meta-analysis 65 recent studies, including 121 effects and 58,368 students, were analyzed. The overall results showed that teacher support was positively correlated with PAEs and negatively correlated with NAEs; the correlation coefficients for these results were both medium. Furthermore, culture, age, and gender moderated these relations.

### The significant correlation between teacher support and students' academic emotions

Meta-analysis results showed a significant positive correlation between teacher support and PAEs and a significant negative correlation between teacher support and NAEs. These results suggest that teacher support is an important mechanism through which teacher can foster students' PAEs and reduce their NAEs (Lawman and Wilson, 2013). These results support a direct effect model, and future studies can test an indirect effect model.

Furthermore, students with difficult learning problems or other problems can seek teacher support as a strategy to improve their PAEs and reduce their NAEs. Furthermore, teacher support is readily accessible on school days and can supplement a student's other interpersonal relationships, especially if the latter are unreliable. In addition, targeted interventions can help students facing difficulties seek out and capitalize on teacher support to improve their learning outcomes.

### Moderation effects

The results also showed that students' culture, age, and gender moderated the relationship between teacher support and students' academic emotions. Specifically, culture and student age moderated teacher support's links with both PAEs and NAEs, and gender moderated teacher support's links with NAEs.

#### Moderating role of culture

Culture moderated the link between teacher support and students' academic emotions, consistent with many prior studies (Jia et al., 2009; Liu et al., 2016). This result suggests that training and interventions should consider cultural aspects, especially cultural differences when adapting training to a new culture. Specifically, the current study obtains the interesting finding that the Western group showed a stronger correlation between

As teacher support had a stronger positive correlation to PAEs among the Western European/American students than the East Asian students, teachers might have a larger impact on enhancing the PAEs of Western European/American students than those of East Asian students. In contrast, teacher support had a stronger negative correlation to NAEs among East Asian students than among Western European/American students, suggesting that teachers might have a larger impact on reducing the NAEs of East Asian students than those of Western European/American students. Future research can examine the mechanisms for these cultural differences.

#### Moderating role of age

Age moderates the relationship between teacher support and students' academic emotions, consistent with past studies (Martínez et al., 2011; Tian et al., 2013; Liu et al., 2016). Further analysis found that the middle school group showed a weaker correlation between teacher support and PAEs and a stronger correlation between teacher support and NAEs than other groups, while the university group obtained a stronger correlation between teacher support and PAEs than other groups. Middle school students are in a psychological weaning period (Huizhen, 2014), and teachers can have a large impact on such vulnerable students with large NAEs. However, their low baseline hinders teachers from sharply increasing their PAEs.

#### Moderating role of gender

Gender moderates the relationship between teacher support and NAEs, with a stronger correlation among female students than among male students; in contrast, gender did not moderate the link between teacher support and PAEs. As the emotional understanding and social skills of females often exceed those of males, female students might express their NAEs to their teachers more effectively than male students do, enabling their teacher support to reduce female students' NAEs more than male students' NAEs. In addition, this finding suggests that similar levels of teacher support may lead to lower NAEs among female than among male students. Considering both age and gender differences in the correlation between teacher support and NAEs, middle school boys emerge as the most vulnerable group, so targeting interventions for them might be especially fruitful.

## Limitations and implications

The current meta-analysis has several limitations. First, only teacher support, involvement, care/caring, warmth, closeness, enthusiasm, and help were selected as indicators of teacher support; other indicators, such as concern, were excluded. Furthermore, the selected indicators may overlap. Second, parallel concerns also apply to indicators of academic emotions. Third, all the studies reviewed examined only direct effects; other studies have found that teacher support can indirectly affects students' academic emotions across other variables as well (Van Ryzin et al., 2009; Sakiz et al., 2012). Therefore, future studies can test for indirect effects, such as whether teacher support indirectly improves academic achievement via academic emotions. Fourth, the current study only considers whether students' culture, age, and gender moderate the relationship between teacher support and students' academic emotions; other variables, such as socio-economic status, can be examined in future studies. Fifth, this study included only English-language articles; future meta-analyses can include studies in other lanugages. Sixth, this meta-analysis was based on cross-sectional studies, so causal relationships cannot be inferred.

## Conclusion

The results of this meta-analysis of 65 studies encompassing 121 effect sizes and 58,368 students revealed that teacher support was significantly correlated with students' academic emotions, and that these relations were moderated by culture, age, and gender. The positive link between teacher support and PAEs was stronger among Western European/American students than among East Asian students. In contrast, the negative link between teacher support and NAEs was stronger among East Asian students than among Western European/American students. The positive link between teacher support and PAEs was strongest among university students and weakest among middle school students. Also, the negative link between teacher support and NAEs was strongest among middle school students and among females.

## Author contributions

HL provided the idea, designed this study and wrote the manuscript, contributed to data collection. YC provided the idea, designed this study and wrote the manuscript, contributed to data analysis. MC contributed to design this study, analysis data and revise paper. All authors approval of the version to be published and agreement to be accountable for all aspects of the work.

### Conflict of interest statement

The authors declare that the research was conducted in the absence of any commercial or financial relationships that could be construed as a potential conflict of interest.
